# The First Lines of the Theory and Practice of Surgery, Including the Principal Operations

**Published:** 1845-11

**Authors:** 


Art. X.—*The First Lines of the Theory and Practice of Surgery, including the Principal Operations.* By Samuel Cooper, Senior Surgeon to university College Hospital, and Professor of Surgery in the same College, etc. *With Notes and Additions.* By Willard Parker, M.D., Professor of Surgery in the College of Physicians and Surgeons in the University of the State of New York, &c., &c. In two volumes. Fourth American from the Seventh London Edition. New York: Samuel S. & William Wood. 1845. 8vo. pp. 1071.

The numerous readers of the former editions of this standard work will be glad to learn that it is again issued in a neat form, and is now accessible to all students of surgery.

A treatise which has passed through seven editions at home and four in this country may well be considered established; but we should not be doing justice to the present edition if we failed to remark that its value has been enhanced by the contributions of the American Editor. Professor Parker is admirably qualified to supply any omissions in a work on surgery. The one before us has been rendered so complete by the author that not much was left to be done by his annotator, but that will be found to correspond in excellence with the text which has been so long a favorite with the profession.

We copy a few of Professor Parker's notes. The first relates to *stricture of the Œsophagus.*

"Stricture of the œsophagus is another disease of which Mr. Cooper makes no mention. This is a disease of the passage, by which its calibre is contracted, and often to such an extent as to cause starvation. It is found at various points in the canal, but most frequently behind the cricoid cartilage, at the junction of the neck with the thorax, and where the œsophagus passes through the diaphragm. It is rarely met with until after middle life, and then is generally cancerous in its character. At times, however, some mechanical cause is concerned in its production, as in the following case:

"A. B., aged 8 years, some two years previous to coming under my care, had swallowed a cent; it seemed not to pass into the stomach at once, and she was never conscious of its passing from her. Almost immediately she began to experience some difficulty in swallowing solid substances, and at length, she could get down but the smallest quantity of liquids. She became very much emaciated, had cough, and death seemed near at hand. I made an attempt to pass a probang, and failed with the smallest. I however succeeded in passing a small bougie, and afterwards the smallest sized probang, through the stricture, and continued to dilate the contraction till at the end of three months, she was able to swallow nearly as well as ever, fattened up, and seemed well. In this case, the stricture must have been produced by inflammation, followed by thickening and contraction of the walls of the walls of the œsophagus, caused by the cent.

"The *symptoms* of this disease are generally well marked, and the case can always be made out, by the aid of the probangs.

"The *prognosis*, in most cases, is unfavorable; as the stricture generally depends upon a scrofulous or cancerous diathesis.

"There are *three* modes of treatment which are resorted to, when anything is attempted to be done.

"1st. *Dilatation*, as in the case given above; and this avails nothing when the disease is malignant; but when the narrowing of the passage is caused by common inflammation, then it succeeds.

"2d. *Caustic* is employed by some surgeons; but I have very little confidence in its use, in the management of strictures in any part of the body.

"3d. We have *Œsophagotomy*, as performed by Dr. John Watson, one of the surgeons of the New York Hospital, in May, 1844, and this I believe to be the only case on record, in which this operation has been performed for the cure of this disease."

We pass by a number of notes to give the following:

"In cases of circocele, when it is cecessary to have recourse to an operation, I have succeeded with the needle and twisted suture. I first separate the vessels from the vas deferens with the thumb and fingers, and then pass a long needle behind them, and apply the thread over the needle as in the hare lip suture. But little pain is experienced, at least for a day or two; if much inflammation follow, the patient should be kept in the recumbent position, diet low, bowels open, and cold applications should be made to the part. I allow the needle to remain until the vessels below it are hard and cord-like.

"I operated in May, 1844, upon a young man 21 years of age, and modified the above operation, by dividing the scrotum over the vessels after the needle was introduced, and then the suture as it was twisted, lay between the lips of the wound, and in this way, the pain from the strangulation of the skin was avoided. My friend Professor Gross of Kentucky, suggested the modification."

Dr. Parker makes some judicious remarks on *fracture of the neck of the thigh bone within the capsular ligament*, and ably controverts the opinion of Sir A. Cooper, that union in such cases never takes place. He thus concludes—

"But enough of this disagreement with others. In the first place, there is a difficulty in diagnosticating with absolute certainty, the exact location of the fracture. We have the transverse fracture entirely within the capsular ligament; and we have it also within and partly without, and between these there is a difficulty of decision.

"Whenever there is any doubt as to whether the fracture be partly without the capsule, we should *always* proceed with a treatment which shall keep the parts in coaptation, and if the constitution be good, we may expect an osseous union.

"When the fracture is diagnosticated to be entirely within the capsule, if the patient be sixty or under, we should *al ways* aim at a union by bone, unless the general health of the patient forbid the confinement. If the patient be over sixty, and the constitution be good, then give him the *chance* of an osseous union. I have in my museum, at the college of Physicians and Surgeons, a perfect specimen of union by bone, in a case of transverse fracture within the capsule, taken from a female who was sixty-four years of age when the accident occurred.

"It often happens, when we have fracture within the capsular ligament, in subjects over sixty, that the bone is so soft and friable as to be crushed into several pieces. In such cases I should avoid the confined mode of treatment, and follow that recommended by Sir Astley Cooper.

"If then, I have simple fracture, at the neck of the thigh bone within the capsule, if the patient be not too old, and the constitution be good, I treat my patient with a view to an osseous union: if, in the event, I fail, I have done my duty.

"After one of these fractures, I allow the patient to remain in a comfortable position for ten or fifteen days, until the more active inflammation has passed, and then I place him in the tight dressings. A roller is applied from the toes to the hip, in order to support the parts; then a broad cushioned belt is carried around the pelvis, and over the trochanters. I commonly use Desault's splint, as modified by the late Dr. Physick; or the same splint as modified by Professor Gibson. The fractured limb is brought down to the same length as the well one, and maintained by the above apparatus in a position corresponding with its fellow. The time required for ossific union varies from six weeks to three months.

"I have been led to these remarks, mainly because of the unfortunate influence which Sir Astley Cooper's opinions, upon this subject, were exerting upon practice, and upon members of the profession. I hold his opinions upon most surgical subjects, as commanding the highest respect, and as at the head of authority; still I cannot believe that a thing *is so*, simply because Mr. Cooper, or any other man, makes the assertion.

"There has been going on, for the last ten years, a most vexatious and malicious law suit in this country, against a highly respectable surgeon, because he treated a patient, a female, forty-three or four years old, for fracture within the capsular ligament, in a manner *not* recommended by Sir Astley Cooper, and succeeded in obtaining an osseous union. In about three months, she was able to walk, both limbs being the same length. When payment for services was demanded, it was refused, upon the ground that there had been no fracture, and that the treatment had been mere sham. The surgeon was sued for malpractice, and Sir Astley Cooper was quoted to prove that if there had been such a fracture, there *could* have been *no* union by bone, and that the limb would necessarily have been shortened and deformed. The patient died some seven years after the accident; and the bone presented ample evidence of fracture within the capsular ligament, and osseous union; and then, and not till then, the poor surgeon, after having been injured in reputation, and ruined in property by the suit was permitted to escape the fangs of a most malicious and unrighteous prosecution."

The last note by the editor refers to the operation for *Strabismus*, and contains minute instructions for the procedure. That operation, after rising with unwonted rapidity into public favor, seems, all at once, to have fallen into disuse. Why is it? Has it failed to remove the blemish, or have the cross-eyed became reconciled to the deformity? Statistics relative to this operation would be instructive.

